# Functionally Characterized Platelet-Rich Plasma Promotes Endothelial Migration and Cutaneous Wound Repair

**DOI:** 10.3390/ijms27209041

**Published:** 2026-10-12

**Authors:** Sanggu Kim, Seongmo Yang, Soochong Kim

**Affiliations:** College of Veterinary Medicine, Chungbuk National University, Cheongju 28644, Republic of Korea; tkdrnfld@naver.com (S.K.); ysm@chungbuk.ac.kr (S.Y.)

**Keywords:** platelet-rich plasma, platelet function, growth factors, HUVEC, skin wound model

## Abstract

Platelet-rich plasma (PRP) has been widely investigated as an autologous therapeutic for tissue regeneration; however, its clinical efficacy remains inconsistent, partly due to variability in PRP preparation protocols. In this study, we established an integrated framework for functional characterization of PRP by integrating platelet recovery, functional integrity, growth factor (GF) release, and regenerative outcomes. PRP prepared using a dual-spin centrifugation protocol at 92× *g* showed significantly higher platelet recovery while preserving functional responsiveness compared to single-spin conditions. PDGF-BB release increased in a time-dependent manner, peaking at 4 h after activation, and was positively correlated with platelet concentration. In an in vitro HUVEC scratch assay, PRP significantly enhanced endothelial cell migration in a platelet concentration-dependent manner. This migratory response was accompanied by increased phosphorylation of ERK and AKT and reduced VE-cadherin expression, indicating modulation of intracellular signaling pathways associated with endothelial cell growth, survival, and migration. In a rat skin wound model, PRP prepared under the selected experimental conditions accelerated wound closure compared with controls. Histological and immunohistochemical analyses revealed enhanced re-epithelialization, increased collagen deposition, increased epithelial cell proliferation, and reduced CD31-positive vascular area, consistent with accelerated vascular remodeling. Taken together, these findings demonstrate that PRP efficacy is governed by multiple interdependent factors. A comprehensive strategy integrating platelet recovery, functional preservation, GF release kinetics, and in vivo and in vitro outcomes provides a rational framework for functional characterization of PRP and may serve as a basis for subsequent translational validation.

## 1. Introduction

Platelets have traditionally been recognized as cellular components that play a primary role in intravascular hemostasis through adhesion, activation, and aggregation at sites of vascular injury. In recent years, however, platelets have attracted substantial attention beyond their conventional hemostatic function as a regenerative therapeutic resource in the form of platelet-rich plasma (PRP), a biologic preparation containing concentrated platelets. PRP has been extensively investigated in a wide range of medical fields, including musculoskeletal disorders, chronic refractory wounds, dentistry, and oral and maxillofacial surgery [1,2]. Its increasing clinical application is largely attributable to the relative simplicity of preparation, favorable biocompatibility, and low incidence of adverse immunologic reactions [3].

The regenerative activity of PRP is largely attributed to growth factors (GFs) and cytokines released following platelet activation. Activated platelets release a broad spectrum of bioactive molecules stored within their granules, including platelet-derived growth factor (PDGF), transforming growth factor-β (TGF-β), vascular endothelial growth factor (VEGF), epidermal growth factor (EGF), and insulin-like growth factor-1 (IGF-1), which contribute to tissue repair through multiple mechanisms [4]. These mediators regulate fibroblast and keratinocyte migration and proliferation, angiogenesis, extracellular matrix synthesis, and re-epithelialization, thereby contributing primarily to the proliferative phases of wound healing. In addition, platelets directly participate in early tissue repair by interacting with exposed collagen at damaged sites, promoting hemostatic plug formation, limiting secondary tissue injury, and providing a provisional matrix for subsequent cellular infiltration [5]. Activated platelets also exert immunomodulatory effects through the release of cytokines and chemokines, facilitating early recruitment of inflammatory cells, followed by the resolution of inflammation and progression toward tissue remodeling [6]. Through these diverse activities, platelets contribute to successive stages of wound healing, from hemostasis and inflammation to proliferation and tissue remodeling.

PRP is commonly prepared by centrifuging whole blood to enrich the platelet-containing fraction, and previous methodological studies have largely sought to increase platelet yield or GF content by modifying centrifugation conditions [7,8]. However, increases in platelet number or soluble mediator concentration alone are insufficient to fully explain the biological efficacy of PRP. Platelets are not passive cellular fragments but highly responsive secretory elements whose regenerative activity depends on multiple functional properties following activation, including the ability to aggregate, granule secretion, preservation of bioactive mediators, and the induction of downstream cellular signaling pathways [9]. Therefore, the biological value of PRP should be assessed not only by quantitative compositional parameters, but also by qualitative functional potency.

Despite growing clinical interest, a universally accepted manufacturing standard for PRP has not yet been established. A major reason for this limitation is the relative lack of comprehensive studies integrating the multiple determinants of PRP performance [7,8]. Previous reports have frequently examined platelet recovery, GF content, cellular responses, or in vivo regenerative outcomes as separate and independent endpoints [6,10]. In practice, however, the therapeutic efficacy of PRP is determined by the complex interaction among preparation conditions, platelet and GF recovery, platelet functionality, activation variables, target-cell responses, and tissue-level regenerative outcomes. Therefore, conclusions derived from isolated parameters may have limited reproducibility and restricted translational applicability. Accordingly, meaningful characterization of PRP requires a systematic framework that elucidates how manufacturing variables influence platelet function and GF release, and how these alterations subsequently affect cellular and tissue regeneration.

In response to these considerations, this study aimed to comprehensively characterize PRP prepared under different experimental conditions using a rat model and to establish a function-oriented framework integrating platelet recovery, functional integrity, GF release, and regenerative outcomes. The rat model provided a controlled preclinical platform for comparing PRP preparation conditions and integrating functional and regenerative outcome assessments. Multiple centrifugation protocols were initially compared to evaluate their effects on platelet recovery and platelet activation competence. Subsequently, the effects of activation time and platelet concentration on PDGF-BB release were examined to characterize biologically relevant application conditions. The functional effects of PRP prepared under different conditions were then evaluated using an in vitro cell migration assay, along with analyses of associated intracellular signaling pathways. Finally, the PRP formulation selected based on the preceding experiments was evaluated in a rat skin wound model by assessing wound closure, histological regeneration, collagen deposition, and expression of proliferative markers. By integrating PRP preparation variables with GF release, cellular responses, and tissue repair outcomes, this study provides an evidence-based framework for functional characterization of PRP in a preclinical setting that may inform subsequent translational validation.

## 2. Results

### 2.1. Complete Blood Count of Rats

**Figure 1:**
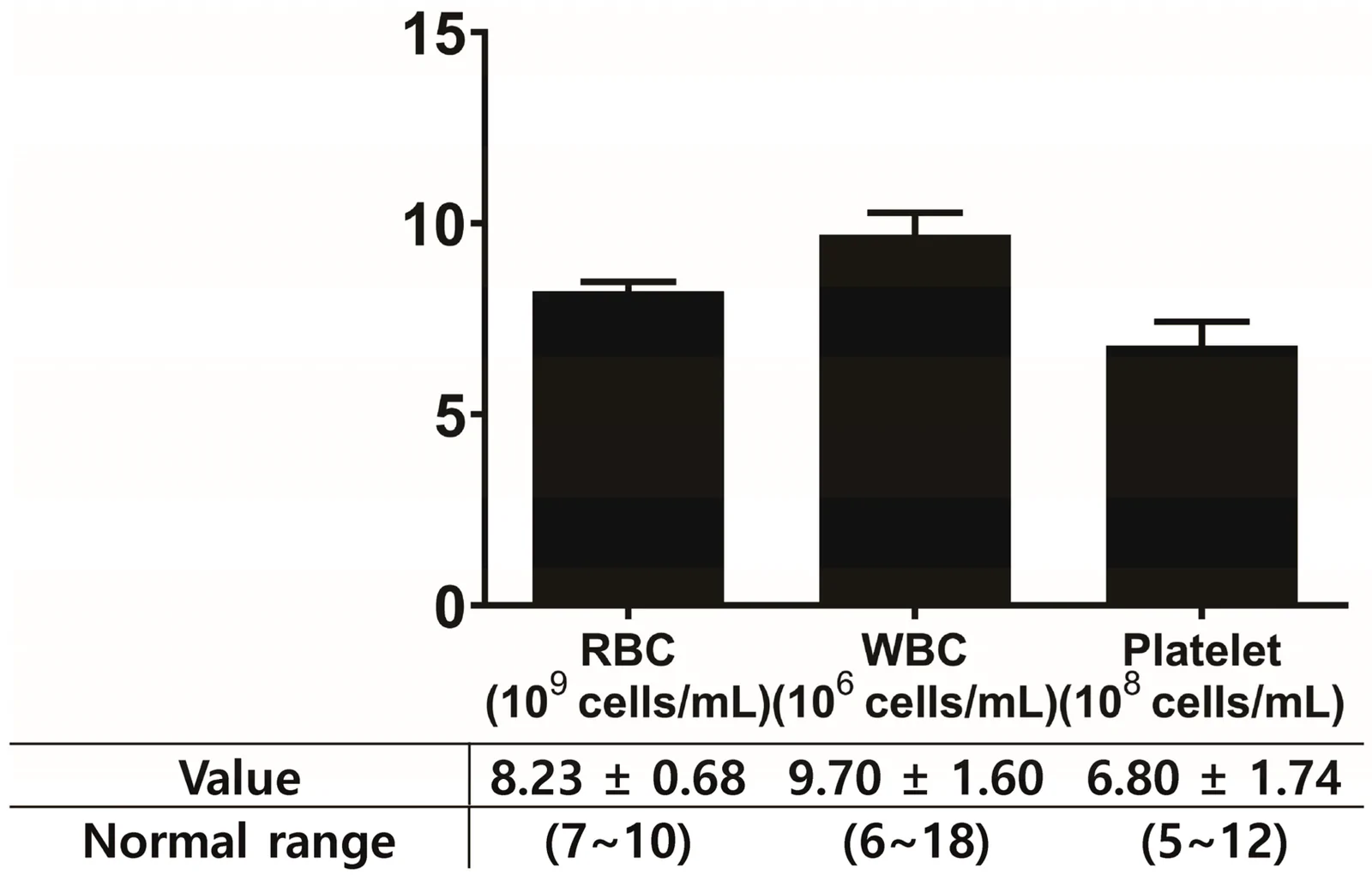
Hematological profiles of rats. Whole blood was collected from healthy rats before PRP preparation, and complete blood count (CBC) analysis was performed. RBC, WBC, and platelet counts were within normal physiological ranges. Data are presented as mean ± SE (*n* = 10).

RBC, WBC, and platelet counts of the rats used in this study (RBC: 8.23 ± 0.68 × 10^9^ cells/mL, WBC: 9.70 ± 1.60 × 10^6^ cells/mL, platelet: 6.80 ± 1.74 × 10^8^ cells/mL) were within the normal physiological ranges (RBC: 7~10 × 10^9^ cells/mL, WBC: 6~18 × 10^6^ cells/mL, platelet: 5~12 × 10^8^ cells/mL) (Figure 1). In addition, no abnormal findings were identified during the general clinical examination, indicating that all animals were in good health prior to blood collection.

### 2.2. Effects of Centrifugation Conditions on Platelet Recovery

**Figure 2:**
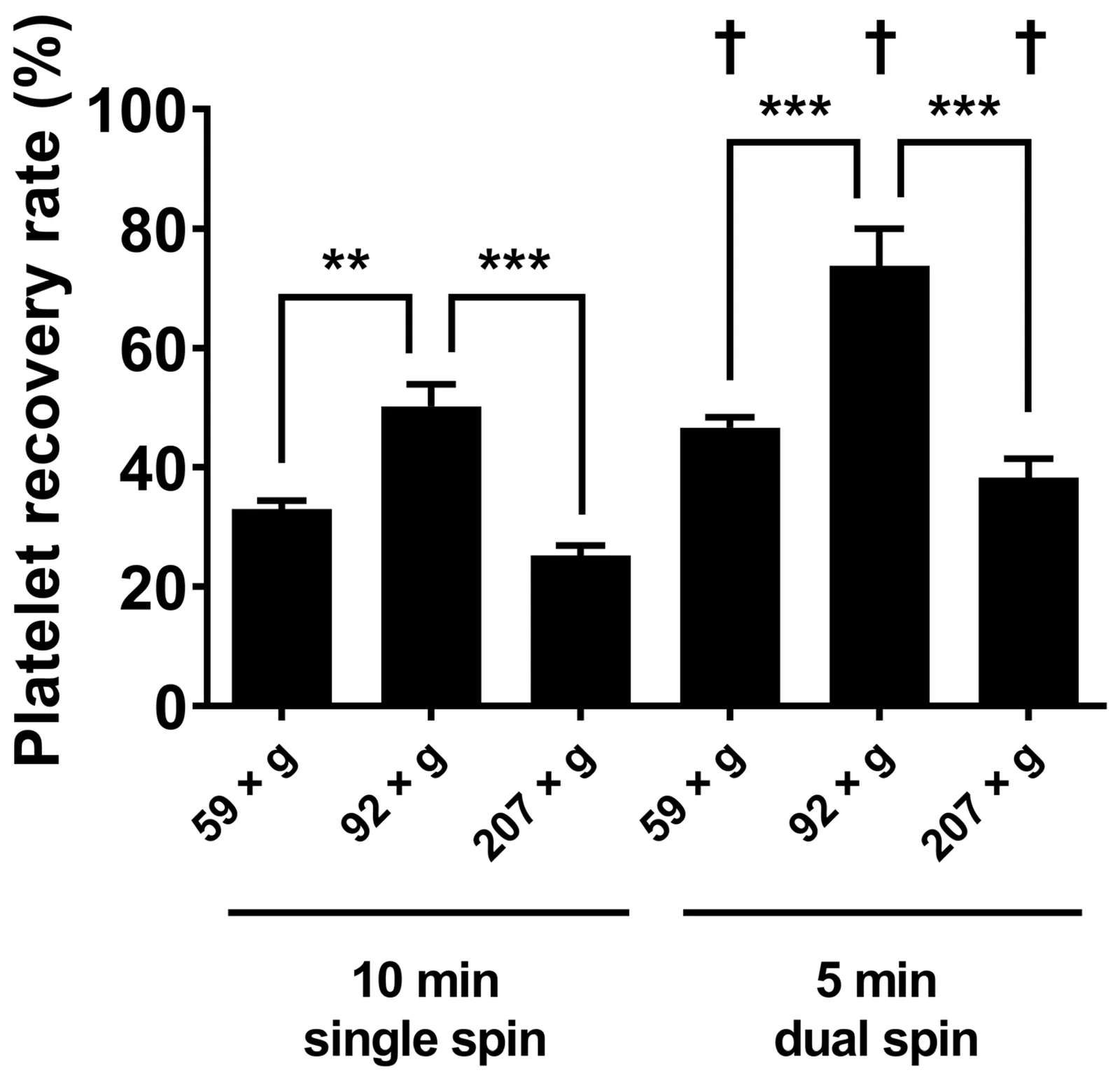
Effects of centrifugation conditions on platelet recovery. Whole blood from healthy rats was subjected to either a single 10 min centrifugation or two consecutive 5 min centrifugation steps at 59, 92, or 207× *g*. Platelet recovery was compared across the experimental conditions (global ANOVA *p* < 0.0001; *n* = 10). Data are presented as mean ± SE. Asterisks denote significant differences between the indicated groups, whereas the obelisk denotes a significant difference from the corresponding single-spin condition at the same relative centrifugal force: **, *p* < 0.01; ***, *p* < 0.001; †, *p* < 0.05.

Efficient platelet recovery is an important consideration in PRP preparation. As shown in Figure 2, platelet recovery was highest at 92× *g*, under a 10 min single-spin centrifugation condition (50.22%). Notably, the dual-spin method using two consecutive 5 min centrifugation steps resulted in greater platelet recovery than the 10 min single-spin at the same relative centrifugal force (73.80%).

### 2.3. Effects of Centrifugation Conditions on Platelet Aggregation and Dense Granule Secretion

**Figure 3:**
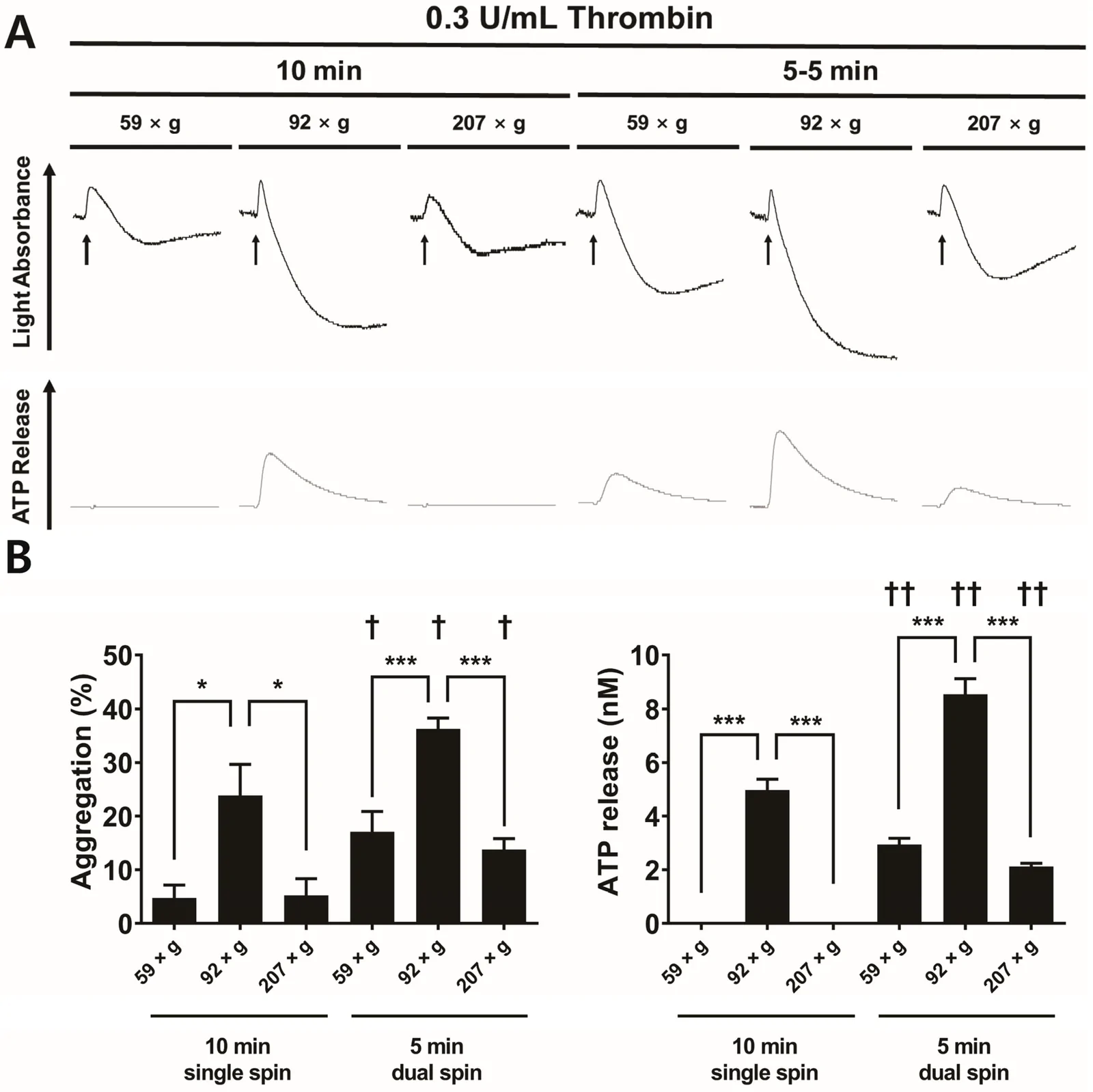
Functional responses of platelets prepared under different centrifugation conditions. (**A**) Washed platelets obtained under each condition were stimulated with 0.3 U/mL thrombin for 3.5 min, and aggregation and dense granule secretion were simultaneously assessed using a Lumi-aggregometer. Arrows indicate the time points of thrombin addition. (**B**) Aggregation and secretion responses shown in (**A**) were quantified (*n* = 10). Data are presented as mean ± SE. Asterisks indicate significant differences between the specified groups, whereas obelisks indicate differences from the corresponding single-spin group at the same relative centrifugal force: *, *p* < 0.05; ***, *p* < 0.001; †, *p* < 0.05; ††, *p* < 0.01.

Since platelet function is an important consideration in the evaluation of PRP preparation conditions, agonist-induced platelet responses were evaluated under each centrifugation condition. Platelet aggregation and dense granule secretion following stimulation with 0.3 U/mL thrombin were measured as indicators of functional integrity. Among the single-spin conditions, the greatest responses to thrombin were observed at 92× *g*, with aggregation of 23.90% and dense granule secretion of 4.98 nM (Figure 3). The corresponding dual-spin protocol further increased the thrombin-induced platelet response (36.29% and 8.54 nM, respectively).

### 2.4. Effects of Platelet Activation Time and Platelet Concentration on PDGF-BB Release

**Figure 4:**
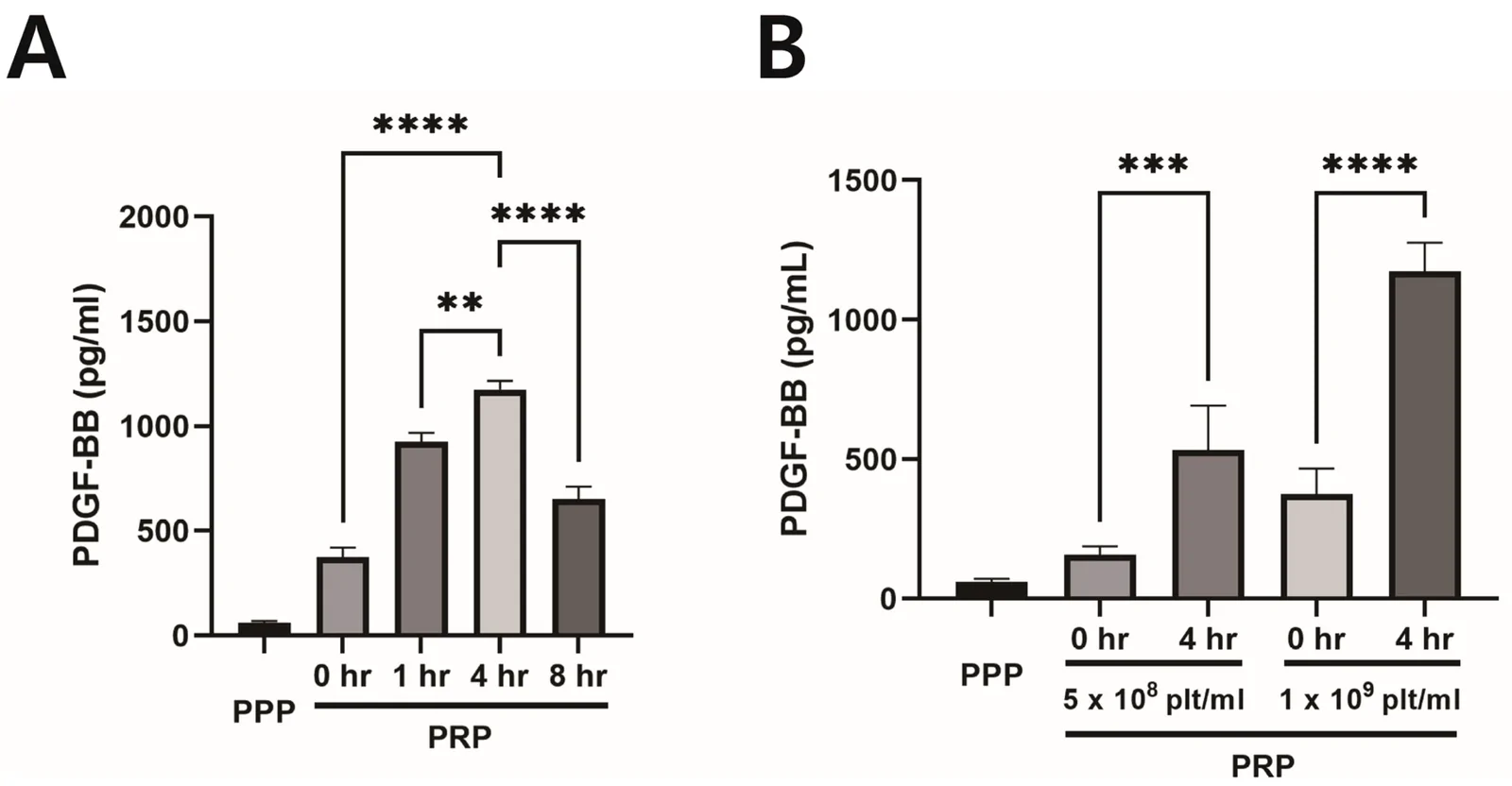
PDGF-BB release from PRP under different activation times and platelet concentrations. (**A**) PRP was prepared using the selected centrifugation condition (92× *g* for 5 min dual-spin), adjusted to 1 × 10^9^ platelets/mL, and activated with 20 mM CaCl_2_ for different durations (0, 1, 4, and 8 h) at 37 °C to compare PDGF-BB release according to activation time (*n* = 6). (**B**) PRP adjusted to either 5 × 10^8^ platelets/mL or 1 × 10^9^ platelets/mL was activated with 20 mM CaCl_2_ for 4 h to compare PDGF-BB release according to platelet concentration (*n* = 6). Data are presented as mean ± SE. **, *p* < 0.01; ***, *p* < 0.001; ****, *p* < 0.0001.

Using PRP prepared under the selected centrifugation condition (92× *g*, 5 min dual-spin), PDGF-BB release was assessed after activation with 20 mM CaCl_2_ at different activation times and platelet concentrations. As shown in Figure 4A, PDGF-BB release progressively increased and reached its maximum after 4 h of activation (1174 pg/mL), followed by a decline at 8 h, suggesting that PDGF-BB release was highest at 4 h among the tested activation times. In contrast, PDGF-BB release increased in proportion to platelet concentration, indicating a positive relationship between platelet concentration and GF release (Figure 4B).

### 2.5. Effects of PRP with Different Platelet Concentrations on HUVEC Migration

**Figure 5:**
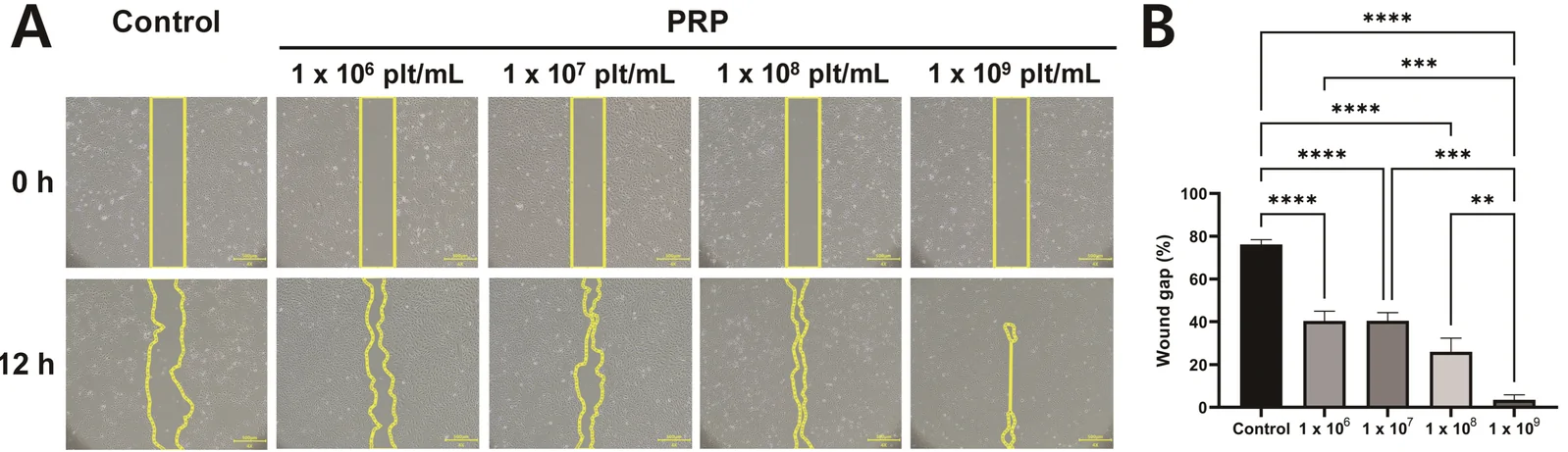
HUVEC migration induced by PRP under different platelet concentrations. (**A**) PRP prepared under the selected centrifugation condition was adjusted to different platelet concentrations (1 × 10^6^, 1 × 10^7^, 1 × 10^8^, and 1 × 10^9^ platelets/mL) and activated with 20 mM CaCl_2_ for 4 h at 37 °C before HUVEC scratch wound assays. Images were captured at the beginning of the assay (0 h) and after 12 h of treatment. Scale bar = 500 µm. (**B**) The residual wound gap was quantified using the images shown in (**A**) (*n* = 5). Data are presented as mean ± SE. **, *p* < 0.01; ***, *p* < 0.001; ****, *p* < 0.0001.

To evaluate the effect of PRP on endothelial cell migration, HUVEC scratch wound assays were performed using PRP with different platelet concentrations. PRP treatment significantly increased scratch closure relative to the saline control, with the response becoming greater as platelet concentration increased (Figure 5). Among the concentrations examined, 1 × 10^9^ platelets/mL resulted in the smallest residual wound gap (3.55%), indicating the greatest scratch closure.

### 2.6. Effects of PRP on ERK/AKT Phosphorylation and VE-Cadherin Expression in HUVECs

**Figure 6:**
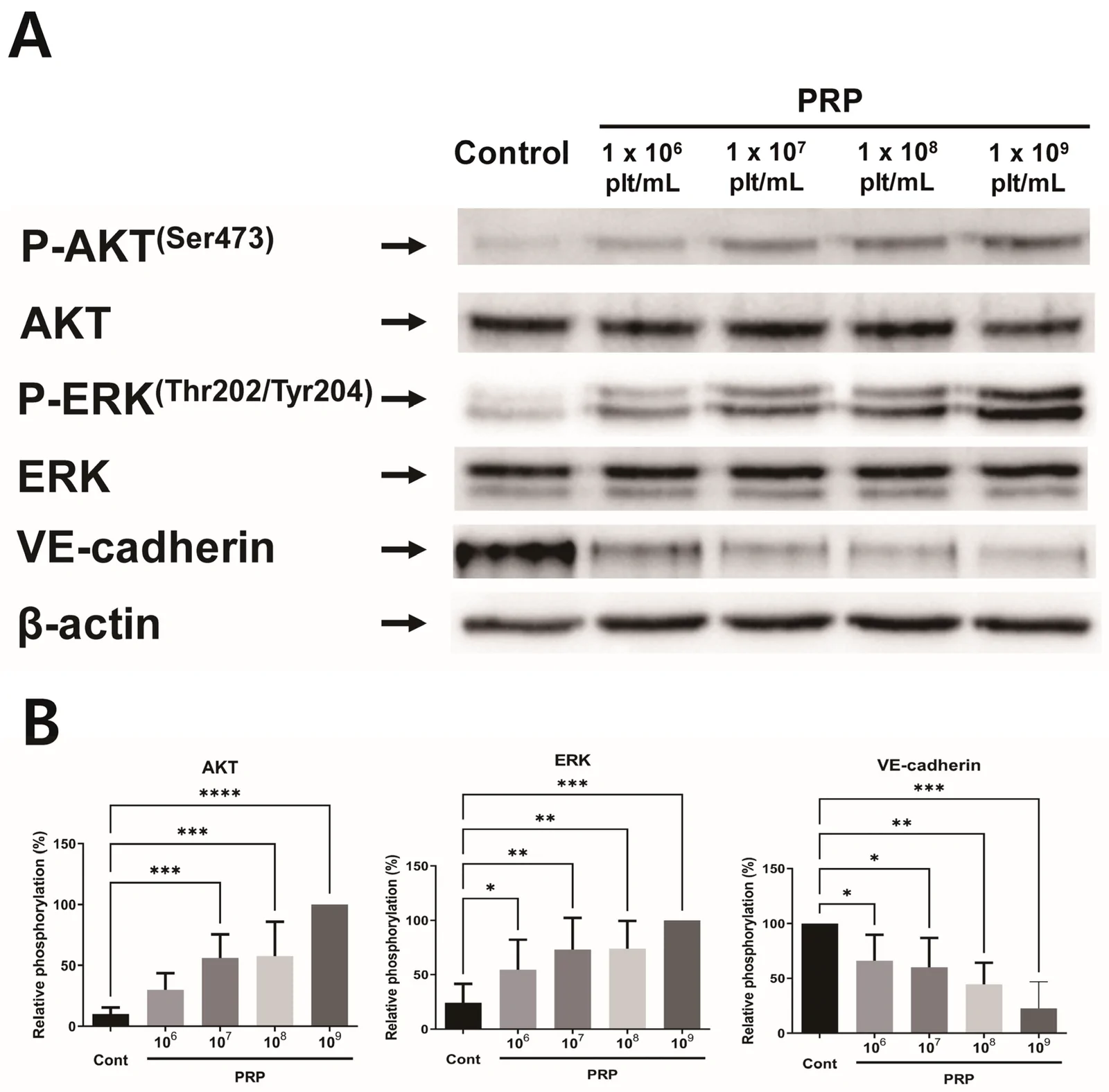
AKT/ERK phosphorylation and VE-cadherin expression in HUVECs under different platelet concentrations of PRP. (**A**) PRP adjusted to different platelet concentrations (1 × 10^6^, 1 × 10^7^, 1 × 10^8^, and 1 × 10^9^ platelets/mL) was applied to HUVECs, and phosphorylation of AKT and ERK and expression of VE-cadherin were measured. (**B**) Relative phosphorylation levels and protein expression were quantified from (**A**) (*n* = 3). Data are presented as mean ± SE. *, *p* < 0.05; **, *p* < 0.01; ***, *p* < 0.001; ****, *p* < 0.0001.

To further investigate signaling responses associated with PRP-induced endothelial cell migration, intracellular signaling responses were examined in HUVECs treated with PRP containing different platelet concentrations. As shown in Figure 6, increasing platelet concentration progressively enhanced the phosphorylation of ERK and AKT, which are involved in multiple cellular processes including growth and survival. In contrast, VE-cadherin expression, a key mediator of endothelial cell migration, was significantly reduced in a platelet concentration-dependent manner.

### 2.7. Effects of PRP on Wound Closure in a Rat Skin Wound Model

**Figure 7:**
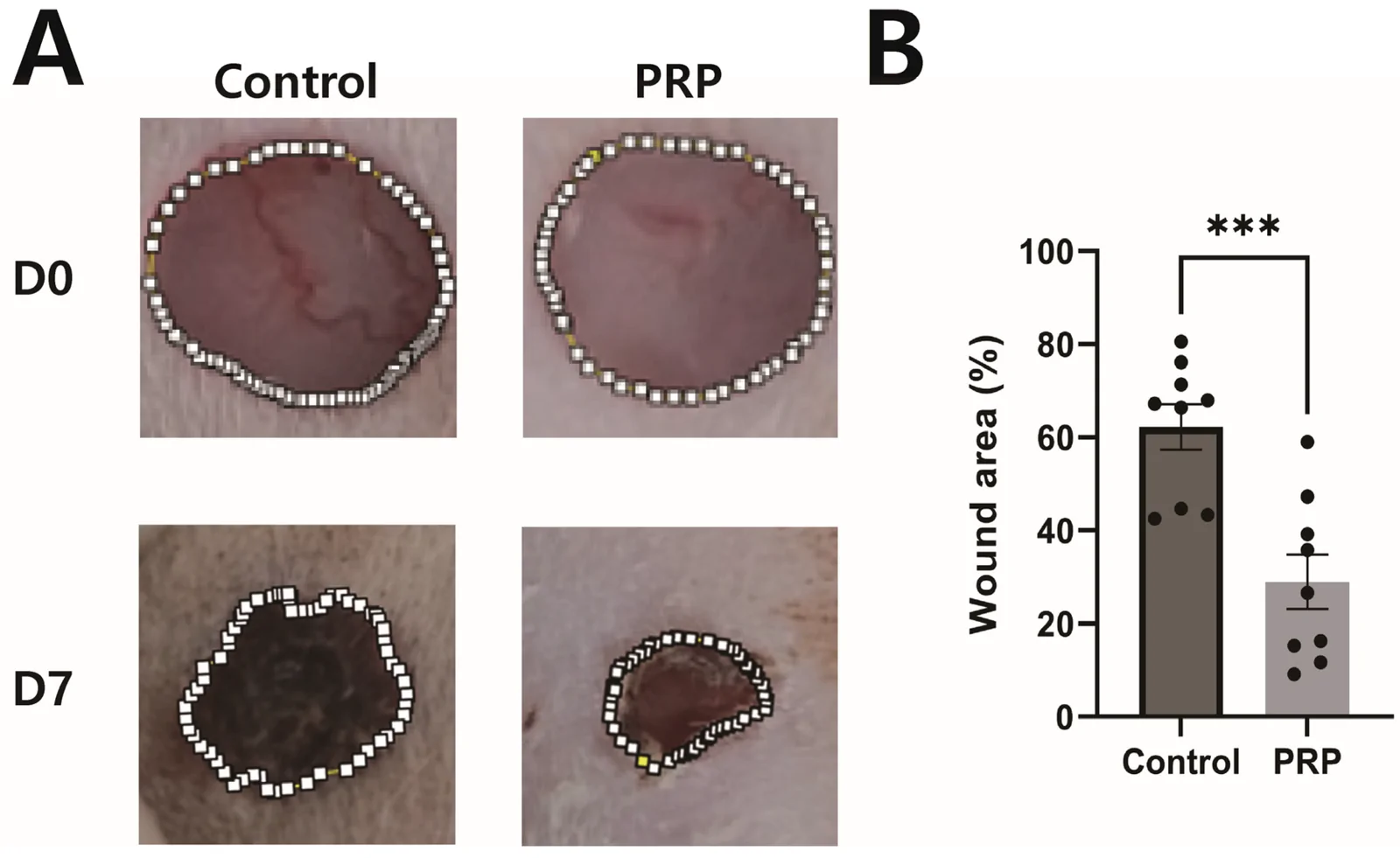
Effects of PRP treatment on cutaneous wound closure in rats. (**A**) PRP was adjusted to 1 × 10^9^ platelets/mL and activated with 20 mM CaCl_2_ for 4 h at 37 °C before in vivo application. Full-thickness 10 mm excisional wounds were generated on the dorsal skin, and each wound received a single application of 100 µL of saline (control) or PRP immediately after wound creation. (**B**) Wound areas were assessed on Days 0 and 7, and the corresponding quantitative analysis is shown (*n* = 9). Data are presented as mean ± SE. ***, *p* < 0.001.

To evaluate the physiological efficacy of PRP, PRP prepared under the selected experimental conditions was applied to a rat full-thickness skin wound model, and wound-healing outcomes were assessed. On Day 7, the remaining wound area was significantly smaller in the PRP-treated group (28.94%) than in the saline-treated control group (62.25%) (Figure 7).

### 2.8. Histological Assessment of PRP Effects on Wound Repair

**Figure 8:**
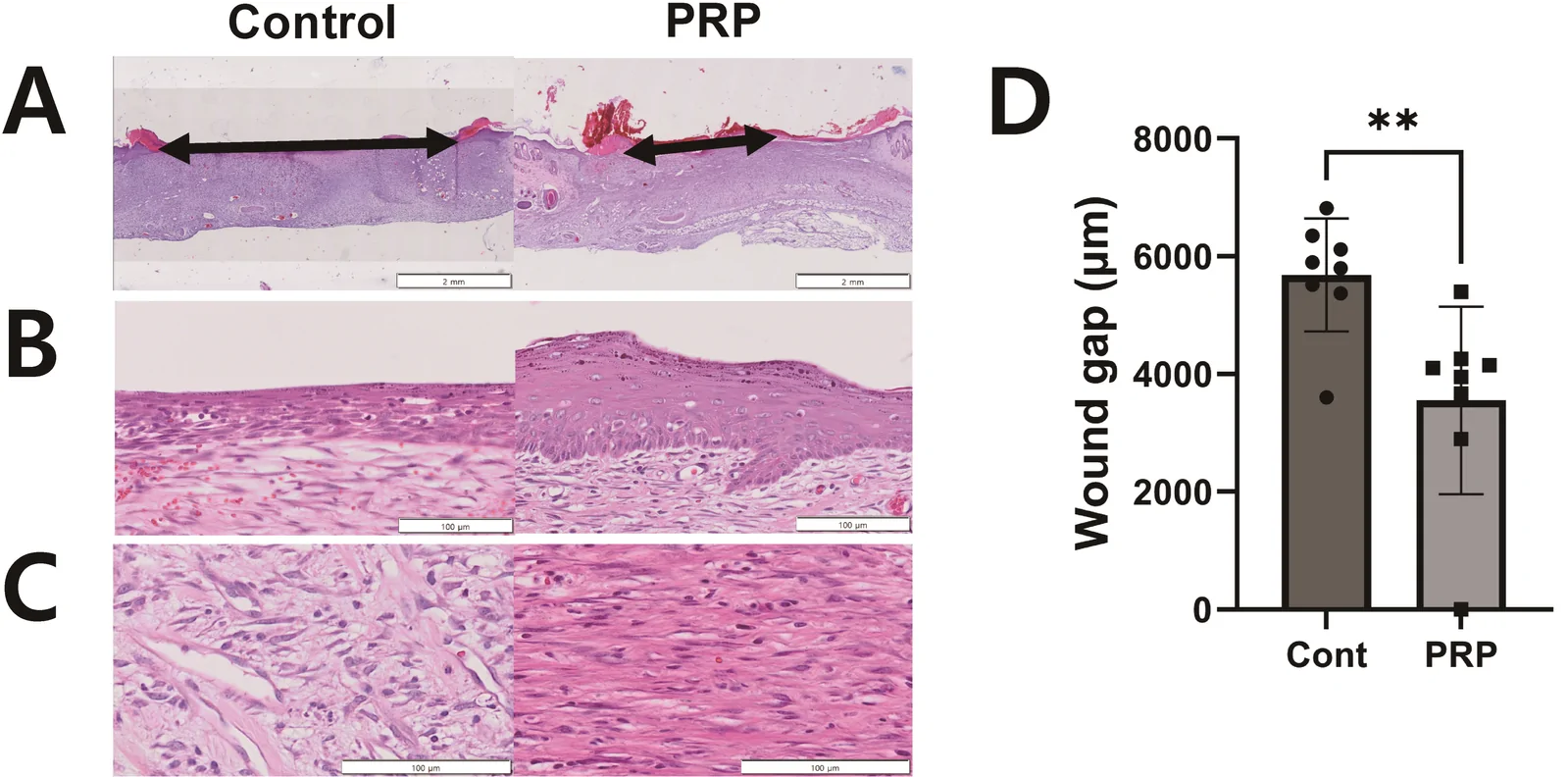
Histological evaluation of rat skin wounds treated with PRP. Wound tissues from saline- or PRP-treated rats were collected on day 7 and processed for hematoxylin and eosin (H&E) staining. (**A**) Representative sections showing the distance between the opposing epithelial margins of the wound (double-headed arrow). Scale bar = 2 mm. (**B**) Representative images of epithelial thickness at one-third of the wound area. Scale bar = 100 µm. (**C**) Representative images of granulation tissue in the dermal wound bed. Scale bar = 100 µm. (**D**) Quantitative analysis was performed from (**A**) (*n* = 8). Data are presented as mean ± SE. ** *p* < 0.01.

Histological evaluation of wound tissues was performed to assess the effects of PRP on tissue regeneration. As shown in Figure 8A, the PRP-treated group (3552 µm) exhibited a significantly reduced wound gap compared with the saline-treated control group (5682 µm). Epithelial thickness measured at one-third of the wound area tended to be greater in the PRP-treated group (Figure 8B). In addition, more mature granulation tissue formation appeared to be present within the dermal wound bed following PRP treatment (Figure 8C). On qualitative examination of the H&E-stained wound sections obtained on Day 7, inflammatory cell infiltration appeared comparable between the control and PRP-treated groups, whereas vascular profiles tended to be less prominent in the PRP-treated group.

### 2.9. Histochemical Assessment of PRP Effects on Collagen Deposition

**Figure 9:**
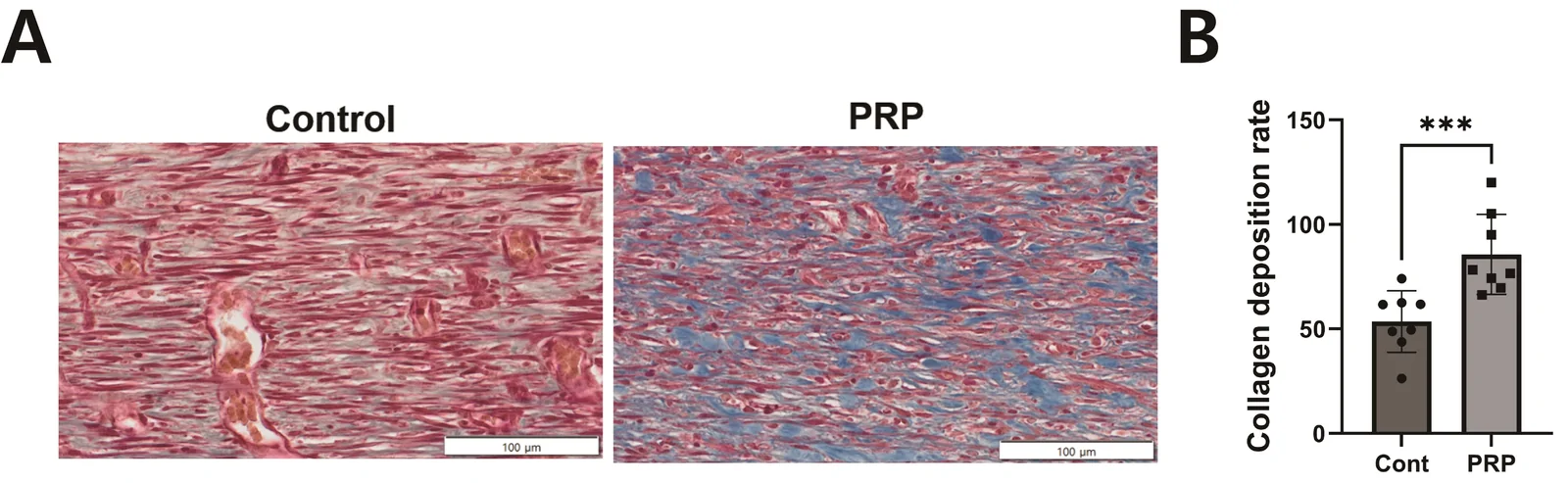
Histochemical evaluation of collagen deposition in rat skin wounds treated with PRP. Wound tissues from saline- or PRP-treated rats were collected on day 7 and stained with Masson’s trichrome. (**A**) Representative images of collagen-positive area in the dermal wound bed. Scale bar = 100 µm. (**B**) Collagen-positive regions (blue staining) were quantified using color deconvolution followed by threshold-based image analysis. Quantitative analysis was performed from (**A**) (*n* = 8). Data are presented as mean ± SE. *** *p* < 0.001.

Collagen accumulation within the wound tissue was examined by Masson’s trichrome staining as an indicator of tissue repair. Quantitative analysis demonstrated a significantly larger collagen-positive area in the PRP-treated group (85.71) than in the control group (53.58) (Figure 9).

### 2.10. Immunohistochemical Assessment of PRP Effects on Epithelial Proliferation

**Figure 10:**
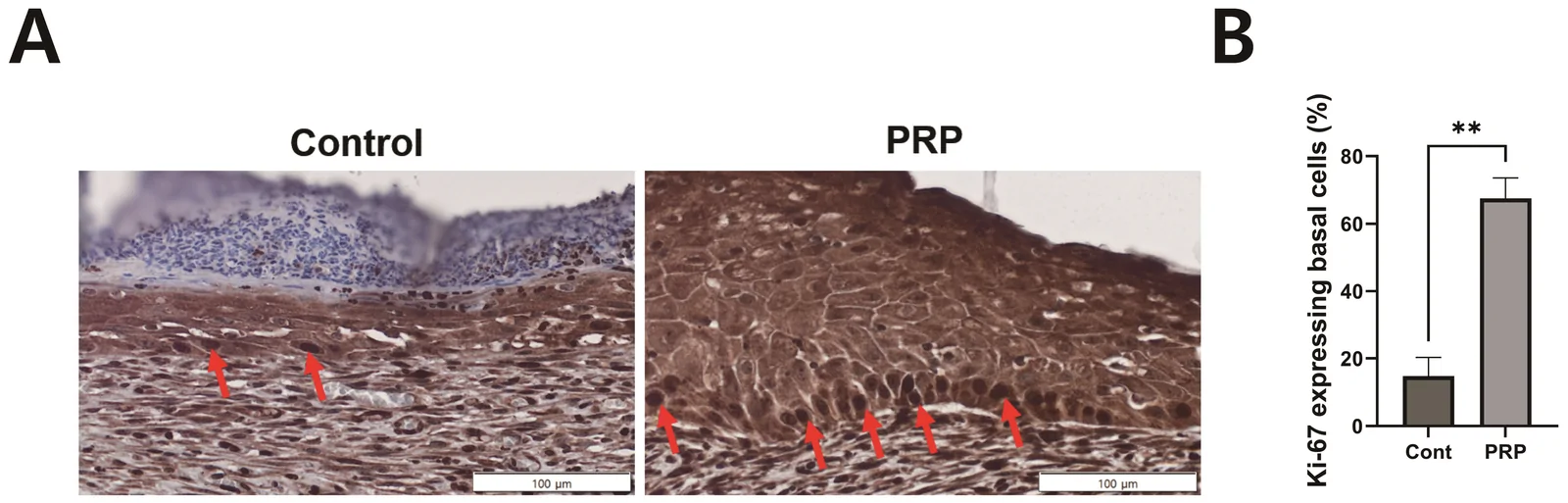
Immunohistochemical evaluation of epithelial proliferation in rat skin wounds treated with PRP. Wound tissues from saline- or PRP-treated rats were collected on day 7 and stained by immunohistochemistry for Ki-67. (**A**) Representative images of Ki-67-positive cells (arrows) at one-third of the wound area. Scale bar = 100 µm. (**B**) The percentage of Ki-67-expressing basal cells was quantified from (**A**) (*n* = 3). Data are presented as mean ± SE. ** *p* < 0.01.

To assess epithelial proliferative activity of PRP during wound healing, immunohistochemical staining against Ki-67 was performed. As shown in Figure 10, the PRP-treated group (67.52%) exhibited a significantly higher proportion of Ki-67-positive basal cells than the control group (14.85%).

### 2.11. Immunohistochemical Assessment of Vascular Remodeling in Mature Granulation Tissue Treated with PRP

**Figure 11:**
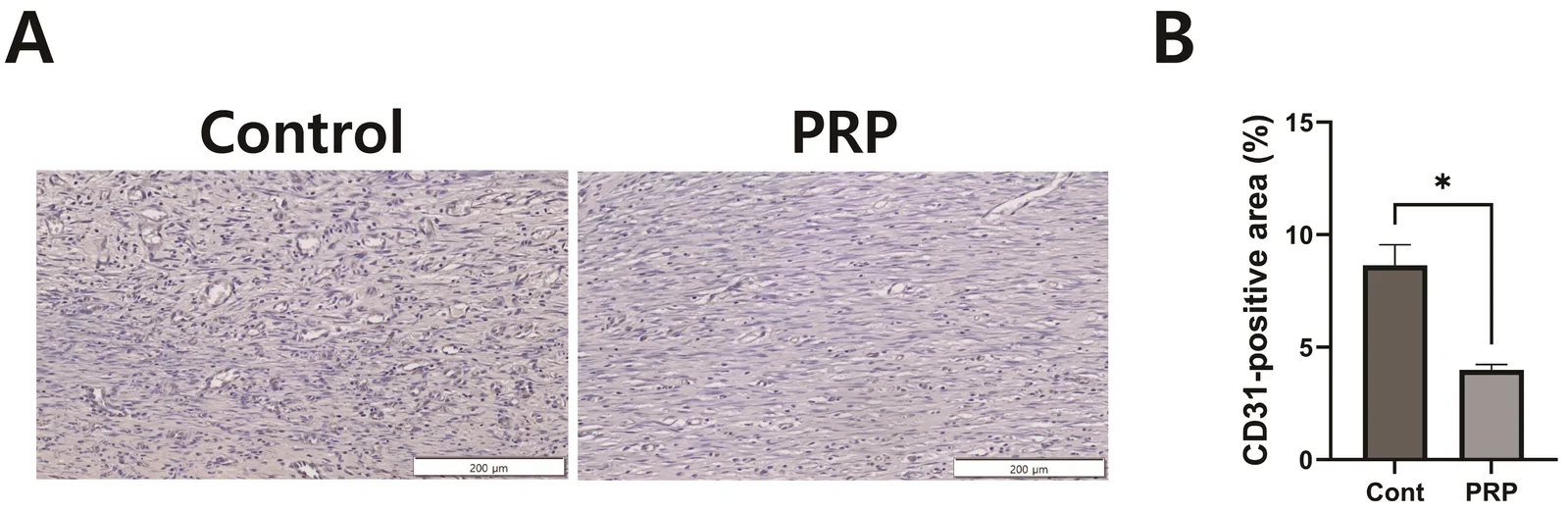
Immunohistochemical evaluation of CD31 expression in rat skin wounds treated with PRP. Wound tissues from saline- or PRP-treated rats were collected on day 7 and stained by immunohistochemistry for CD31. (**A**) Representative images of CD31 immunoreactivity within the granulation tissue of the wound bed. Scale bar = 200 µm. (**B**) CD31-positive regions (brown staining) were quantified using color deconvolution followed by threshold-based image analysis. Quantitative analysis was performed from (**A**) (*n* = 3). Data are presented as mean ± SE. * *p* < 0.05.

To assess the effect of PRP on vascular remodeling during wound healing, immunohistochemical staining for CD31 was performed. As shown in Figure 11, mature granulation tissue from the PRP-treated group (4.00%) exhibited a significantly lower CD31-positive area compared to immature granulation tissue from the control group (8.65%).

## 3. Discussion

PRP has been widely investigated as an autologous biological therapeutic for tissue regeneration due to its low immunogenicity and its ability to provide a concentrated reservoir of platelet-derived mediators involved in wound healing [2]. Beyond its primary role in hemostasis, PRP exerts regenerative effects through the release of multiple GFs that regulate cellular proliferation, migration, inflammation resolution, angiogenesis, and extracellular matrix remodeling [11]. However, the biological efficacy of PRP remains inconsistent across studies, largely owing to variations in preparation methods, activation protocols, and heterogeneity in platelet composition [7,8]. Furthermore, although an integrated approach is essential to fully elucidate the complex physiological functions of platelets, most previous studies have focused on individual endpoints, such as GF content, in vitro cellular responses, in vivo regenerative outcomes, or clinical effect, thereby limiting a comprehensive understanding of platelet functionality [6,10]. In the present study, we systematically evaluated multiple factors influencing PRP performance, including centrifugation parameters, platelet functional integrity, activation time, platelet concentration, and regenerative efficacy in both in vitro and in vivo models. Our findings suggest that comprehensive functional characterization of PRP should extend beyond platelet recovery alone and incorporate functional preservation, GF release kinetics, and biologically relevant application conditions.

Platelet recovery represents an important initial consideration in PRP preparation. Insufficient recovery may limit the availability of regenerative mediators, particularly in settings where blood volume is restricted. In the present study, the dual-spin centrifugation protocol yielded a higher platelet recovery rate than the single-spin approach under comparable conditions. This finding is consistent with our previous study in a canine model, in which dual-spin was shown to improve platelet enrichment [12]. Interestingly, the centrifugation condition selected in the present rat study (92× *g*) differed from that identified in our previous canine study (364× *g*). This discrepancy likely reflects species-specific hematological characteristics, such as differences in blood cell size, hematocrit, and plasma viscosity [13]. This interspecies difference also indicates that centrifugation conditions identified in one species should not be assumed to be directly transferable to another. In addition, technical factors, including tube size, sample volume, and sedimentation distance, may have substantially influenced the efficiency of centrifugation. In our experimental setup, 1 mL of whole blood was processed in a 1.7 mL microtube, which may have altered the effective sedimentation path and consequently influenced the centrifugation condition associated with greater platelet recovery. L. Piao et al. proposed a theoretical model predicting platelet recovery based on centrifugation parameters, blood volume, composition, and tube size, suggesting that increasing blood volume, using tubes with larger cross-sectional areas, and diluting samples could enhance recovery by extending the sedimentation distance [13]. However, such modifications may necessitate higher centrifugal force or prolonged centrifugation time, thereby increasing shear stress on platelets [14]. Therefore, platelet recovery should not be considered in isolation, but rather balanced with the preservation of platelet functionality and overall biological efficacy.

Importantly, the therapeutic efficacy of PRP depends not only on platelet concentration but also on the functional integrity of platelets. In the present study, platelets isolated under the selected centrifugation condition exhibited enhanced agonist-induced aggregation and dense granule secretion, indicating that platelet responsiveness was well preserved. Excessive centrifugal force may impose increased shear stress on platelets, leading to premature activation, membrane damage, granule depletion, and ultimately reduced responsiveness [14,15,16]. In contrast, the reduced platelet function observed under lower centrifugal force, despite lower shear stress, remains unclear. This may be associated with incomplete separation, contamination with other blood components, or altered recovery of platelet subpopulations, which could ultimately affect overall platelet functional capacity. These findings suggest that the selection of PRP preparation conditions should consider both platelet quantity and functional competence rather than platelet recovery alone.

GF release was strongly influenced by platelet activation time. In our study, PDGF-BB release increased gradually following CaCl_2_-induced activation, peaked at 4 h, and subsequently declined. This pattern indicates that platelet-derived GF release is not an immediate or continuously increasing process, but rather a regulated, time-dependent event. The initial increase likely reflects progressive platelet activation and granule secretion, whereas the subsequent decline may result from proteolytic degradation or reduced protein stability [17,18]. These findings have important practical implications, as PRP is often applied either immediately after activation or following empirically determined incubation periods.

PRP exerts regenerative effects not only through its hemostatic function but also by releasing multiple GFs that promote tissue repair. To evaluate the direct cellular effects of platelet functionality and GF release, in vitro application to cultured cells is necessary. It has been widely reported that the application of PRP to HUVECs enhances both proliferation and migration [6]. Consistent with previous findings, our study demonstrated that PRP enhanced HUVEC migration, with a greater migratory response observed as platelet concentration increased. The most pronounced migratory response was observed when PRP at a concentration of 1 × 10^9^ platelets/mL was applied. At the molecular level, phosphorylation of ERK and AKT increased in parallel with platelet concentration. ERK promotes cellular proliferation by regulating transcription factors such as c-Fos and c-Jun [19,20]. Similarly, AKT, a central component of the PI3K/AKT pathway, enhances proliferation via mTOR activation and mediates anti-apoptotic effects by upregulating Bcl-2 and suppressing pro-apoptotic factors, including caspase-3 and Bad [21,22,23]. In addition, VE-cadherin is known to transiently decrease during cell migration due to the temporary weakening of intercellular junctions and subsequent internalization from the plasma membrane [24]. In our study, phosphorylation of ERK and AKT, which are involved in multiple cellular processes including growth and survival, increased in proportion to higher platelet concentrations in PRP. Conversely, VE-cadherin expression, which decreases during cell migration, was further reduced with higher platelet concentrations, supporting an effect of PRP on endothelial cell migratory signaling. Nevertheless, excessively high platelet concentrations do not always enhance cell activity and have been reported to exert adverse effects on proliferation and migration. Since PRP has been shown to promote proliferation and migration in various cell types, including alveolar bone cells, keratinocytes, fibroblasts, and Schwann cells, the appropriate platelet concentration should be determined depending on the specific cell type and experimental context [25,26,27,28].

Skin wounds represent one of the most common forms of injury encountered in daily life and can range from mild to severe conditions such as diabetic ulcers, burns, radiation-induced damage, and chronic infections, which are often difficult to treat and significantly impair quality of life [29]. To extend our experimental findings to a more complex biological environment, we evaluated PRP treatment in a rat skin wound model. In our study, the PRP-treated group exhibited accelerated re-epithelialization and enhanced granulation tissue formation. This regenerative effect was further supported by the observed increases in collagen deposition and basal cell proliferation, both of which are essential for epidermal regeneration. These results are consistent with previous reports demonstrating that PRP accelerates epithelial regeneration, angiogenesis, and granulation tissue maturation in cutaneous wounds [6].

On qualitative examination of the H&E-stained wound sections obtained on Day 7, inflammatory cell infiltration appeared comparable between the control and PRP-treated groups, whereas vascular profiles tended to be less prominent in the PRP-treated group. Consistent with this histological observation, quantitative CD31 immunohistochemistry demonstrated a significantly lower CD31-positive area in the PRP-treated group than in the control group on Day 7, indicating accelerated vascular remodeling in the PRP-treated group. These findings should be interpreted in the context of the temporal dynamics of inflammation and angiogenesis during wound healing. In rat cutaneous wounds, the inflammatory response has been reported to reach its maximum level around Day 5 and subsequently decline after Day 7, whereas the proliferative phase begins around Day 3 and is most active around Days 5–7. VEGF expression similarly increases through Day 7 and subsequently decreases as healing progresses [30]. A time-course analysis of rat cutaneous wound healing further demonstrated that vascular volume peaks and subsequently declines during tissue remodeling [31]. Moreover, the dense neocapillary network generated during the proliferative phase is progressively pruned as the wound enters the resolution and remodeling phases [32]. PRP may accelerate this temporal progression. In a previous PRP-treated cutaneous wound model, prominent granulation tissue accompanied by inflammatory and vascular infiltration was observed at Day 5, whereas the granulation tissue was largely absorbed by Day 7 [6]. Therefore, the comparable inflammatory cell infiltration observed between groups at Day 7 in the present study may reflect convergence of the inflammatory response at this later stage of healing. Taken together with the accelerated wound closure, increased collagen deposition, and enhanced epithelial proliferation observed in the PRP-treated group, the reduced CD31-positive area on Day 7 suggests that PRP treatment may promote vascular remodeling and maturation of granulation tissue during wound healing, rather than reduced angiogenesis throughout the healing process.

Several limitations of the present study should be considered. First, the PRP preparation conditions identified in this rat model should not be interpreted as universally transferable across species. The difference between the centrifugation condition selected in the present rat study and that identified in our previous canine study highlights the influence of species-specific hematological characteristics and experimental factors on platelet recovery and function. Accordingly, although the rat model provides a controlled platform for functional characterization of PRP preparation conditions, it has inherent limitations for directly defining preparation parameters applicable across species or clinical settings. Second, histological and immunohistochemical evaluation of wound healing was performed at a single time point (Day 7), limiting direct assessment of the temporal dynamics of inflammation and angiogenesis. Although CD31 immunohistochemistry provided a quantitative assessment of wound vascularization at Day 7, the single-time-point design does not directly establish the preceding temporal pattern of angiogenesis or vascular regression. In addition, ERK/AKT activation and VE-cadherin expression were not directly evaluated in wound tissues; therefore, the signaling responses observed in HUVECs should not be interpreted as direct evidence of identical molecular changes in vivo. Finally, no direct proliferation assay was performed in HUVECs, and the observed ERK/AKT activation should therefore be interpreted as growth- and survival-associated signaling rather than direct evidence of enhanced endothelial proliferation. Future studies incorporating multiple sampling time points, serial evaluation of vascular remodeling, additional inflammatory analyses, tissue-level molecular analyses, and validation in other species and human samples will be required to further establish the temporal mechanisms and translational applicability of this functional characterization framework.

Collectively, our findings highlight the regenerative potential of PRP in both epidermal and dermal layers and indicate the importance of carefully selecting and functionally evaluating PRP preparation and application conditions.

## 4. Materials and Methods

### 4.1. Materials

Apyrase (type V), Mitomycin C, and Prostaglandin E1 (PGE1) were purchased from Sigma-Aldrich (St. Louis, MO, USA). Thrombin was purchased from Tocris Bioscience (Bristol, UK). Anti-phospho-AKT (Ser473), Anti-phospho-ERK (Thr202/Tyr204), Anti-AKT, Anti-ERK, Anti-beta-actin, Anti-VE-cadherin, and Anti-rabbit IgG antibodies were purchased from Cell Signaling Technology (Beverly, MA, USA). Luciferin-luciferase was purchased from Chrono-Log (Havertown, PA, USA). Anti-Ki-67 and anti-CD31 antibodies were purchased from Abcam (Waltham, MA, USA). Penicillin-streptomycin was purchased from BANDIO Bio Science (Pocheon, Republic of Korea). RIPA lysis buffer, phosphatase inhibitor, and protease inhibitor were purchased from ATTO Corporation (Tokyo, Japan). Chemiluminescence substrate and SuperBlock blocking buffer were purchased from Thermo Fisher Scientific (Rockford, IL, USA). Human umbilical vein endothelial cells (HUVECs) and endothelial cell basal medium were purchased from PromoCell (Heidelberg, Germany). Eosin and Harris hematoxylin were purchased from BBC Biochemical (McKinney, TX, USA). Masson’s trichrome stain kit was purchased from Polysciences (Warrington, PA, USA). ImmPACT™ DAB substrate and Vectastain Elite ABC-HRP kit were purchased from Vector Laboratories (Newark, CA, USA). All additional chemicals were of reagent grade.

### 4.2. Animals

8-week-old male Sprague-Dawley rats (Koatech, Pyeongtaek, Republic of Korea) were obtained and acclimated for at least one week prior to the experiment. Animals were housed under controlled environmental conditions (22 ± 2 °C, 50 ± 10% humidity) with free access to food and water. All animal experiments were performed with approval from the Institutional Animal Care and Use Committee of Chungbuk National University (CBNUA-2076-23-02). Ten rats were used exclusively as blood donors for PRP preparation, whereas a separate cohort of nine rats was used for the in vivo wound-healing experiment.

No formal a priori sample size calculation was performed. Sample sizes were determined based on experimental feasibility and assay-specific requirements and were comparable to those reported in previous PRP-related studies employing similar experimental approaches [33,34,35,36]. All reported n values represent independent biological replicates rather than technical replicates. No predefined inclusion or exclusion criteria were established for animals, experimental units, or data points. No animals or data points were excluded from the analyses. The exact sample size used for each analysis is provided in the corresponding figure legend.

### 4.3. PRP Preparation

Blood samples were collected from donor rats by terminal cardiac puncture under 2–3% isoflurane anesthesia (Hana Pharm, Seongnam, Republic of Korea). A total of 9 mL of blood was drawn into a 10 mL syringe preloaded with 1 mL of 3.8% sodium citrate, which served as an anticoagulant by coating the inner surface of the syringe. Following blood collection, the donor animals were not allowed to recover from anesthesia and were not subsequently included in the in vivo wound-healing experiment. Collected blood was divided into 1 mL aliquots in 1.7 mL microtubes (Axygen, Union City, CA, USA). Blood samples were centrifuged using a fixed-angle rotor microcentrifuge (Corning LSE High-Speed Microcentrifuge, Corning Incorporated, Corning, NY, USA; radius = 8.24 cm) at 59× *g* (800 rpm), 92× *g* (1000 rpm), or 207× *g* (1500 rpm), using either a 10 min single-spin or 5 min dual-spin (two-time immediate sequential repetition of the same centrifugation condition for 5 min) [12]. After centrifugation, the upper plasma fraction was aspirated. The obtained supernatant was centrifuged at 405× *g* (2100 rpm) for 10 min to pellet the platelets, and the platelet pellet was resuspended with PPP or Tyrode’s buffer (138 mM NaCl, 2.7 mM KCl, 2 mM MgCl_2_, 0.42 mM NaH_2_PO_4_, 5 mM glucose, and 10 mM HEPES; pH 7.4) containing 0.05 unit/mL apyrase to prepare PRP or washed platelets, respectively. For quantitative analysis, the concentrations of RBCs, WBCs, and platelets were measured using a Celltac Alpha MEK 6550 complete blood count analyzer (Nihon Kohden, Tokyo, Japan).

### 4.4. Platelet Aggregation and Secretion

Washed platelets adjusted to a concentration of 2 × 10^8^ platelets/mL were used to evaluate agonist-induced platelet aggregation and secretion. The assays were performed using a Lumi-aggregometer (Chrono-Log, Havertown, PA, USA) at 37 °C under continuous stirring at 900 rpm for 3.5 min. Aliquots of washed platelets (250 µL) were stimulated with thrombin, and changes in light transmission were recorded. Platelet secretion was assessed by measuring ATP release after addition of luciferin-luciferase at a final concentration of 0.2 µmol/L. Results were expressed as maximal aggregation (%) and ATP release (nmol/L).

### 4.5. PDGF-BB Quantification

Platelet samples at concentrations of 5 × 10^8^ or 1 × 10^9^ platelets/mL were activated by the addition of 20 mM CaCl_2_ and incubated at 37 °C for 0, 1, 4, or 8 h. Following incubation, the samples were stored at −80 °C until analysis. After thawing, the samples were centrifuged at 3000× *g* for 10 min to obtain the supernatant. The collected supernatant was diluted 20 times with phosphate-buffered saline, and the concentration of PDGF-BB was determined in duplicate using a Canine PDGF-BB ELISA kit (Ray Biotech, Norcross, GA, USA) according to the manufacturer’s instructions.

### 4.6. HUVEC Scratch Wound Healing Assay

HUVECs were seeded at a density of 1 × 10^5^ cells/well in 6-well culture plates and maintained in endothelial cell basal medium supplemented with 1× penicillin-streptomycin and a supplement mix (5% FBS, 0.5 ng/mL EGF, 2 ng/mL bFGF, 90 µg/mL heparin, 1 µg/mL hydrocortisone, and 1 µg/mL ascorbic acid). When cells reached 90–100% confluence, the medium was replaced with endothelial cell basal medium containing 10% of the supplement mix to induce a starvation condition, and a uniform linear scratch wound was created using a sterile 1 mL pipette tip. After scratching, cells were treated with PRP containing different platelet concentrations (0, 1 × 10^6^, 1 × 10^7^, 1 × 10^8^, and 1 × 10^9^ platelets/mL). Wound size was measured after 12 h of incubation, and the degree of wound closure was compared among the experimental groups.

### 4.7. Western Blot Analysis

HUVECs were incubated with PRP for 30 min for analysis of ERK and AKT phosphorylation or 12 h for analysis of VE-cadherin expression. For migration-related Western blot analysis, cells were additionally treated with 10 µg/mL mitomycin C for 2 h after starvation to inhibit cell proliferation. At the indicated time points, cells were lysed with 100 µL of RIPA buffer containing protease and phosphatase inhibitors (1000:1:1, *v*/*v*/*v*). Plates were incubated on ice for 15 min with gentle agitation and centrifuged at 12,000× *g* for 15 min at 4 °C to remove insoluble debris. Protein concentrations were determined using a BCA protein assay kit (iNtRON Biotechnology, Seongnam, Republic of Korea). Lysates were mixed 1:1 with 2× Laemmli sample buffer containing 5% β-mercaptoethanol (Bio-Rad Laboratories, Hercules, CA, USA) and denatured at 100 °C for 10 min prior to Western blotting.

Protein samples were separated on a 10% SDS polyacrylamide gel and transferred onto a 0.2 µm nitrocellulose membrane using the Trans Blot Turbo Transfer System (Bio-Rad, Hercules, CA, USA). The membranes were blocked by incubation with SuperBlock blocking buffer to prevent nonspecific binding. After blocking, the membranes were incubated overnight at 4 °C with anti-phospho-AKT (Ser473), anti-phospho-ERK (Thr202/Tyr204), anti-AKT, anti-ERK, anti-VE-cadherin, and anti-beta-actin antibodies. Following primary antibody incubation, the membranes were washed and probed with anti-rabbit IgG antibody for 1 h at room temperature. Immune reactivity was then visualized with a chemiluminescent substrate and detected with an iBright 1500 (Invitrogen, Waltham, MA, USA).

### 4.8. In Vivo Rat Skin Wound Model

A separate cohort of nine rats was used for the in vivo wound-healing experiment. Rats were anesthetized with 2–3% inhaled isoflurane. The dorsal hair was shaved and the skin was disinfected with 70% ethanol prior to wound creation. Four full-thickness excisional skin wounds were created on the dorsum of each rat using a 10 mm biopsy punch (Acuderm Inc., Fort Lauderdale, FL, USA). Within each animal, two wounds were assigned to the saline control and the remaining two wounds were assigned to PRP treatment. Immediately after wound creation, the wound beds were treated once with 100 µL of saline or PRP containing 1 × 10^9^ platelets/mL after 4 h of activation and then covered with Tegaderm (3M, St. Paul, MN, USA). No additional PRP treatment was administered during the 7-day observation period. Animals were housed individually to prevent wound interference and monitored daily for signs of distress or infection. Wound areas were measured on days 0 and 7 using ImageJ (version 1.53t; National Institutes of Health, Bethesda, MD, USA). The remaining wound area (%) was calculated as [(Day 7 wound area)/Day 0 wound area × 100].

Humane endpoints included severe or persistent distress, marked reduction in food or water intake, severe wound infection, and marked deterioration in general condition. Animals were monitored daily, and no animals reached the predefined humane endpoints.

### 4.9. Histological and Immunohistochemical Analysis

Skin wound tissues collected on postoperative day 7 were fixed in 10% neutral buffered formalin. After fixation, tissues were processed routinely and embedded in paraffin to prepare formalin-fixed paraffin-embedded blocks. Each sample was sectioned at a thickness of 5 µm and mounted on silane-coated glass slides (Muto Pure Chemicals Co., Ltd., Tokyo, Japan).

Paraffin sections were deparaffinized and stained with hematoxylin and eosin (H&E) or Masson’s trichrome. After staining, sections were dehydrated, cleared in xylene, and mounted with coverslips (Marienfeld Superior, Lauda-Königshofen, Germany).

For immunohistochemical staining, rehydrated sections underwent antigen retrieval and were blocked with 5% goat serum. Sections were incubated with an anti-Ki-67 or anti-CD31 antibody at 4 °C overnight. After washing with PBS, slides were incubated with a diluted biotinylated goat anti-rabbit secondary antibody for 30 min at room temperature, followed by incubation with an avidin–biotin complex reagent for 30 min at room temperature. Immunoreactivity was visualized using ImmPACT™ DAB substrate. Sections were counterstained with hematoxylin for 1 min, dehydrated, and mounted with coverslips.

Histologically and immunohistochemically stained tissue sections were scanned using a slide scanner (Olympus Slideview VS200, Olympus, Tokyo, Japan). To evaluate wound closure, the distance between the opposing epithelial margins of the wound was measured under low magnification and compared among groups. Re-epithelialization was compared based on epithelial thickness at one-third of the wound length. H&E-stained sections were also qualitatively examined for inflammatory cell infiltration and the prominence of vascular profiles within the wound bed. Collagen deposition was evaluated using Masson’s trichrome staining as an indicator of granulation tissue formation and maturation. Epithelial cell proliferation was assessed by quantifying Ki-67-positive basal cells. Wound vascularization was assessed by quantifying CD31-positive areas within the granulation tissue of the wound bed.

For Masson’s trichrome staining analysis, blue-stained collagen regions were isolated using the color deconvolution plugin in ImageJ software. Extracted regions were thresholded and staining intensity was quantified. Ki-67 expression was quantified at one-third of the wound region by calculating the proportion of basal cells showing positive nuclear immunoreactivity. For CD31 analysis, DAB-positive regions were isolated using the color deconvolution plugin in ImageJ software. Extracted regions were thresholded and the CD31-positive area was quantified.

### 4.10. Statistical Analysis

All statistical analyses were performed using GraphPad Prism (version 9.1; GraphPad Software, San Diego, CA, USA). Data are presented as mean ± standard error (SE). Paired Student’s *t*-test was used for two-group matched comparisons, and repeated-measures one-way analysis of variance followed by Bonferroni’s post hoc test was used for multiple matched comparisons. Normality was assessed using the Shapiro–Wilk test. A two-sided *p* value < 0.05 was considered statistically significant.

## 5. Conclusions

Numerous previous studies have attempted to standardize PRP preparation by maximizing platelet recovery, and extensive efforts have also been made to evaluate the regenerative effects of PRP in various experimental and clinical models. However, these studies have often been conducted independently, without integrating PRP preparation parameters with functional and biological validation. In contrast, the present study provides a comprehensive framework that links PRP preparation parameters with platelet functional activity, GF release kinetics, in vitro cellular responses, and in vivo wound healing outcomes. The preparation conditions selected within the experimental range tested in this study, comprising dual-spin centrifugation, activation with 20 mM CaCl_2_ for 4 h, and a platelet concentration of 1 × 10^9^ platelets/mL, provided a favorable balance among platelet recovery, functional preservation, and biological efficacy.

In conclusion, this study demonstrates that the efficacy of PRP is determined by multiple factors, including platelet recovery, platelet functional preservation, activation kinetics, and platelet concentration. A function-oriented approach that integrates these variables provides a more comprehensive framework for functional characterization of PRP than platelet count-based evaluation alone. This integrated framework may provide a basis for subsequent translational validation across different species and experimental settings.

## Data Availability

The data presented in this study are available from the corresponding author upon reasonable request.
